# Effects of Aquatic Therapy for Children with Autism Spectrum Disorder on Social Competence and Quality of Life: A Mixed Methods Study

**DOI:** 10.3390/ijerph18063126

**Published:** 2021-03-18

**Authors:** Javier Güeita-Rodríguez, Anna Ogonowska-Slodownik, Natalia Morgulec-Adamowicz, Mar Lledó Martín-Prades, Juan Nicolás Cuenca-Zaldívar, Domingo Palacios-Ceña

**Affiliations:** 1 Research Group of Humanities and Qualitative Research in Health Science of Universidad Rey Juan Carlos (Hum&QRinHS), Department of Physical Therapy, Occupational Therapy, Rehabilitation and Physical Medicine, Universidad Rey Juan Carlos, 28922 Madrid, Spain; domingo.palacios@urjc.es; 2 Faculty of Rehabilitation, Jozef Pilsudski University of Physical Education in Warsaw, 00-968 Warsaw, Poland; anna.ogonowskaslodownik@awf.edu.pl (A.O.-S.); natalia.morgulec@awf.edu.pl (N.M.-A.); 3 Pediatric Brain Damage Unit, Nuestra Señora del Carmen, 46024 Valencia, Spain; marlledomarpra@gmail.com; 4 Rehabilitation Unit, Hospital de Guadarrama, 28440 Madrid, Spain; nicolas.cuenca@salud.madrid.org; 5 Department of Physical Therapy, Universidad Francisco de Vitoria, 28223 Madrid, Spain

**Keywords:** autism, aquatic program, multimethod research, integration approaches

## Abstract

Autism Spectrum Disorder (ASD) is a constellation of social deficits and repetitive sensory-motor behaviours. Aquatic therapy (AT) may be effective in improving the social interactions and behaviours in children with ASD. The objective of this study was to evaluate the effects of an AT program on social competence and quality of life and to understand participant’s experiences related to the intervention by obtaining qualitative data. A mixed methods intervention study was conducted among 6 children with ASD and their parents, with two research phases in a concurrent embedded design (an aquatic intervention as the quantitative design and a qualitative design in second step). The intervention and qualitative design followed international guidelines and were integrated into the method and reporting subheadings. Significant improvement was observed in the physical competence (*p* = 0.026) and important improvements in school functioning and aquatic skills, with no adverse events. Qualitative findings described: the meaning of AT intervention, patterns of behaviour and activities changes, social communication and social interaction. The aquatic intervention showed positive results for the social and physical competence, with elements of discordance, expansion, and confirmation between quantitative and qualitative results.

## 1. Introduction

Autism spectrum disorders (ASD) are multifactorial disorders of neurodevelopment that appear in the early stages of life [1]. In 2018, it was estimated that the overall prevalence of ASD among Spanish children was 15.5 per 1000 in preschoolers and 10 per 1000 in school-age children [2]. They are diagnosed by a significant impairment in social communication and repetitive sensory and motor behaviours [1]. In addition to concerns about social communication and behaviour, studies have addressed the fact that children with ASD have great motor difficulties. Provost et al. noted that at least 60% of young children with ASD could meet the criteria for early intervention by health professionals based on their motor difficulties alone [3]. Motor problems reported in children with ASD include clumsy gait, poor muscle tone, balance difficulties, poor motor control and manual dexterity, and difficulties with praxis and movement planning [4,5]. McPhillips et al. concluded that children with ASD are at risk for clinically significant motor impairments [6].

Gernsbacher et al. showed that the acquisition of motor skills is relevant to everyday skills such as language and communication, founding a strong association between early oral and manual motor abilities and later speech fluency [7]. Other authors stated that motor skills are also related to emotional and social functioning [8,9], and the way children play and interact [10]. Even walking problems and postural asymmetry have been shown to have an effect on schooling and socialization of these children [11]. According to Sutera et al., better motor control is associated with a decrease in the severity of non-motor symptoms of autism in adulthood [12]. Overall, previous studies suggest that the performance of motor skills in children with ASD may have significant long-term effects in counteracting the developmental cascade in the social, emotional and behavioural domains [8,13,14] beyond just the acquisition of motor competence [15]. Kasari et al. found that teaching functional motor abilities enables participation in social activities, increasing opportunities for interaction and thus producing highly positive effects for other problems [16].

Previous evidence suggest that the teaching of motor skills would encourage important changes in behaviour (such as imitation, social attention, speech and communication). Among the types of intervention identified to improve both motor skills and social behaviours is aquatic therapy (AT). AT is a therapeutic modality in an enriched environment, with considerable advantages due to the water properties, such as hydrostatic pressure, water temperature, viscosity, and buoyancy [17]. Based on the literature, AT appears to be particularly beneficial for children with ASD, who need strong sensory stimulation. It involves vigorous movement in contact with and against water pressure, and the intense sensory stimulation received may result in an overall calming effect and improve the children’s ability to interact and communicate with others. Mortimer et al. in their systematic review, point out that AT is a promising treatment method for social interactions and behaviour, especially while using the Halliwick concept. The purpose of the Halliwick concept is to apply a progressive program in particular, focusing on individuals with physical and/or learning difficulties, to participate in water activities, to move independently in water, and to swim. It comprises four phases: mental adjustment to the water, rotations, control of movement, and movement in the water [18]. Some authors specifically added learning strategies to the Halliwick swimming intervention [19,20,21,22] with different feedback chosen (continuous reinforcement, unique opportunity and social reinforcements). Few studies reported improvements of water skills and water orientation [23,24,25]. In terms of physical condition and social behaviour, statistically significant differences were also found with respect to the control groups [24,25,26]. Parental and children participation and satisfaction were measured by Fragala-Pinkham, who reported high levels of satisfaction with the aquatic program [23]. Mills et al. showed that AT may enhance behaviours impacting mental health and well-being of children [27]. Quality of life was improved in the sub-domains of physical function, emotional and social aspects, and school aspects [28]. In all the interventions using learning strategies, the objectives were to achieve certain aquatic skills or the performance of water games [19,20,21,22]. All studies described earlier [19,20,21,22,23,24,25,26,27,28] were conducted solely on children with ASD, while Pan’s research was only one comparing children with ASD with their siblings [25].

Experimental interventions using enriched environments for the treatment of diseases should be analysed to determine their efficacy and safety, as well as the experience, perspective and satisfaction of the users [29]. Both quantitative (e.g., intervention effectiveness) and qualitative (patient acceptance or refusal of therapy) aspects need to be studied in relation to the treatment approach in different intervention settings [30]. Mixed method research combines the strengths of quantitative and qualitative research to gain a deeper understanding [31]. This methodology has previously been used in research involving health services, such as program evaluation, implementation of innovative interventions [31], clinical issues, and health care decision-making, including a supplementary qualitative component within pilot studies of complex interventions [30]. Previous studies have described the use of mixed methods in children with neurodevelopmental disorders (not in ASD), combining analysis of the effects of experimental interventions with qualitative research on the impact of various treatments [32,33].

The Qualitative Research in Trials (QUART) study [29] reported on how qualitative health research had been used in trials and identified ways to maximize the value of a trial in providing evidence of treatment efficacy. QUART provided the way for using mixed studies for qualitative analysis of a new intervention in efficacy studies (quantitative component). This approach determines whether an intervention is carried out as intended, describes the implementation processes, generates an understanding of why the intervention worked or failed, and demonstrates whether the effectiveness of the therapy is promoted or limited in real-world situations. Similarly, new treatments must also be investigated under real clinical conditions, where patients and their environment (away from the clinical setting) do not necessarily adhere to ideal experimental criteria [34]. Qualitative analyses can be conducted before, during or after the quantitative evaluation of the intervention [35]. Before applying the treatment to larger samples, the study protocols should be progressively tested to describe their efficacy, safety, and patient acceptance [29]. These issues are especially important in complex patients, such as children with ASD, who often have multiple comorbidities, take multiple medications, and have varying levels of functional impairment [36].

Therefore, the current study aimed to use a concurrent integrated design to investigate the use of an AT intervention for children with ASD. To the best of our knowledge, this is the first study that attempts to describe the impact of AT intervention with learning strategies on children with ASD using mixed methods, to evaluate the intervention from clinical and parental perspectives. The study included both quantitative and qualitative objectives. The objective of the quantitative design was twofold: to implement an AT program, using learning strategies specifically designed by the research team for children with ASD and to analyse its effects on perceived competence and social acceptance, aquatic skills, and quality of life. The qualitative design objective was to explore and describe the experiences regarding AT among parents of children with ASD, as well as to assess emotions and to describe the polarity through parents’ narratives (acceptance or rejection) of this way of treatment.

## 2. Materials and Methods

**Figure 1:**
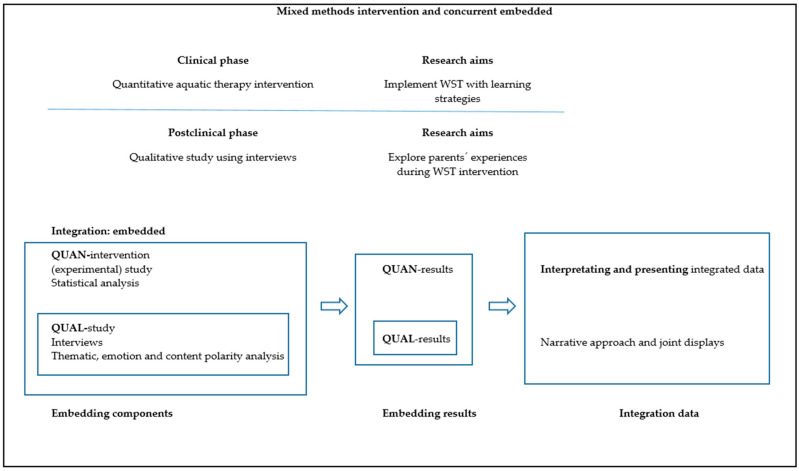
Mixed methods design and embedded integration. Quan, quantitative; Qual, qualitative; WST, Water Specific Therapy.

A mixed-method intervention study design with a qualitative component was applied. Within a concurrent integrated design [35], in the first step of data analysis, the quantitative methodology was used, while in the second step, qualitative methodology was used. Qualitative data were collected after the AT intervention to elucidate possible mechanisms and explain the quantitative results (participants’ experience, intervention-related improvements, and possible facilitators and barriers) [35]. Table 1 summarizes the methodology used in the research process. Quantitative and qualitative data were integrated at the method level by embedding one into the other [35], and at the level of interpretation and reporting through narrative and joint presentations [37]. Figure 1 shows the design of the mixed methods and embedded integration.

The integration phase of the study elucidates the understanding of the relationships between the quantitative and qualitative components [35]. The aim of this integration phase is to balance the respective strengths and weaknesses of the methods to optimize the performance of the various complementary sources of evidence [35].

In this study, we followed the Best Practices for Mixed Methods Research in the Health Sciences from the National Institutes of Health [38] and the guidelines provided in Good Reporting of a Mixed Methods Study [39]. The Template for Intervention Description and Replication checklist (TIDieR) [40] was used in the quantitative intervention phase (see Table S1).

We have followed the guidelines for qualitative studies established in the Standards for Reporting Qualitative Research [41] and the Consolidated Criteria for Reporting Qualitative Research [42]. In addition, we followed the criteria for ensuring the reliability of qualitative research as proposed by Guba and Lincoln [43,44]. The various techniques performed and the application procedures used to monitor reliability are described in Table S2.

### 2.1. Quantitative Intervention Phase Design

A non-randomized clinical intervention was conducted in the first quantitative research step, involving children with ASD.

#### 2.1.1. Sampling and Participants

All patients were recruited from the Formato Integral Centre (Valencia, Spain) with a non-probabilistic sampling of non-consecutive cases. The study participants had to meet the following inclusion criteria: (a) age: 6–12 years; (b) medical diagnosis of Autism Spectrum Disorder; (c) participation in continued treatment with AT for at least three months prior to the beginning of the study (prescribed techniques need time to cause mental adjustment and solid confidence); (d) informed consent signed by the legal guardians. The exclusion criteria were the coexistence of other disorders, including genetic disorders and psychiatric conditions.

#### 2.1.2. Intervention

The TIDieR checklist [40] was followed (Table S1). The Water Specific Therapy (WST)–Halliwick sessions included four different phases in order to influence the internal processes associated with distorted experiences and to lead to relatively permanent changes in the capacity to perform activities [45]. These phases were (1) Entrance ritual: stimulating the approach to the new environment. (2) Mental adjustment: controlling and dosing different types of sensory inputs. (3) Learning phase: design of specific tasks using strategies that support learning. (4) Exit ritual: calm down and make a connection with the transfer out of the water.

**Figure 2:**
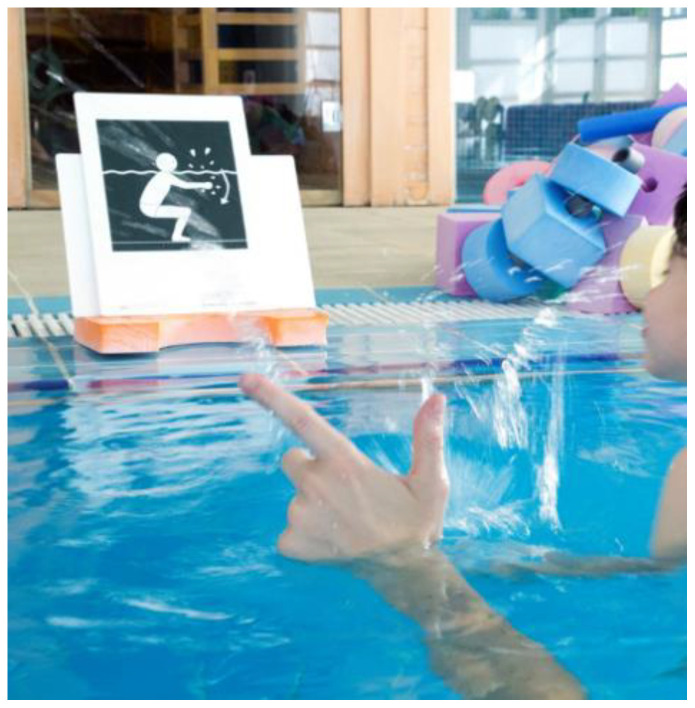
Example of visual prompting and attentional cues used in aquatic sensorimotor task.

WST sessions were carried out at the swimming pool of Formato Integral Centre during individual AT sessions lasting 60 min twice a week over seven months, and provided by the same physical therapist, who is specialist in AT. This WST intervention was part of the intervention programs at the Formato Integral Centre. The main goal of this WST intervention was to enhance the sensorimotor performance, cognitive potential, enjoyment and social aspects among children and youth with ASD, as a feasible alternative to conventional physical therapy. Various aquatic sensorimotor tasks with learning strategies were used (Figure 2).

#### 2.1.3. Data Collection and Outcome Measures

The Pictorial Scale of Perceived Competence and Social Acceptance for Young Children (PSPCSA) for the first and second grades was administered to measure the perceived competence and social acceptance. Psychometric properties of PSPCSA are considered acceptable [46]. Twenty-four items in four subtests were analysed: cognitive competence; peer acceptance; physical competence; maternal acceptance; with higher points meaning higher level of competence or acceptance.

To assess the child’s ability to adapt to the aquatic environment and related functional ability, the Water Orientation Test Alyn version 1 (WOTA 1) was administered. The WOTA 1 scale is reliable and valid for assessing mental adaptation and aquatic function in children with disabilities [47]. This test consists of 15 items with a score from 0 to 3, depending on the ability to perform the functional task in the aquatic environment.

The Pediatric Quality of Life Inventory (PedsQL) parent-report (23-item version) was administered to measure health-related quality of life. PedsQL has been shown to have strong psychometric properties in a sample of individuals with developmental disabilities, including individuals with ASD [48]. Four multidimensional scales were analysed: physical health; emotional, social, and school functioning. The items in each of the dimensions are scored from 0 to 4 according to the problems the child has had in his or her daily life and the difficulty the child has had in the last month in carrying out the task in question. Zero points are equivalent to no problem and 4 points mean problems almost always or the partial inability to carry out such activity (extreme difficulty). Next, the items are reverse scored and linearly transformed to a 0–100 scale (0–100, 1–75, 2–50, 3–25, 4–0), so that higher scores indicate better health-related quality of life.

All assessments were performed by the same physical therapist trained in the use of the measurement instruments and not related to the intervention received by the subjects. The outcome measures were administered at the beginning (pre) and at the end (post) of the intervention.

#### 2.1.4. Data Analysis

The statistical analysis of the data obtained was carried out using IBM SPSS Statistics (version 21, Armonk, NY, USA). A descriptive analysis of the entire sample and the study variables of the PSPCSA, WOTA1 and PedsQL scales included: mean, standard deviation, minimum and maximum. Due to lack of data normal distribution (the Kolmogorov–Smirnov test) and small sample size, non-parametric, statistical tests were used. The Wilcoxon signed-rank test for related samples was used to compare variables at the beginning and at the end of intervention. Additionally, the effect size was determined by calculating the r according to the formula presented by King et al. [49]. Values of r > 0.50, >0.30 and >0.10 were typically considered to represent large, moderate, and small meaningfulness of results, respectively. The statistical analysis was calculated with a 95% confidence level. A value of *p* < 0.05 was established to determine statistical significance.

### 2.2. Qualitative Phase Design

We conducted a qualitative exploratory study [43,44].

#### 2.2.1. Sampling and Participants

Purposeful sampling methods were used based on their relevance to the research question rather than clinical representativeness [50]. All parents were recruited from the AT trial. Recruitment took place when the patients completed their WST intervention in the swimming pool at the Formato Integral Centre. Parents were included in the qualitative phase if their child met the inclusion criteria of our study and agreed to participate. The sampling process was based on the information power criteria established by Malterud et al. [51]. Information power indicates that the more information the sample has that is relevant to the study, the fewer participants are needed. For this reason, the same participants who were recruited for the intervention in the quantitative phase were included in qualitative phase. No participants withdrew from the study.

#### 2.2.2. Data Collection

Data collection consisted of semi-structured interviews, based on a question guide, designed to obtain information regarding specific topics of interest [43] (Table S3). The question guide was developed based on a prior literature review [52,53] along with the researchers’ experience [43]. The interviews were tape-recorded and transcribed verbatim. Overall, 295 min of data collection was recorded. All interviews were held either at the Formato Integral Centre or at the participants’ home, depending on parents’ preference. Researcher field notes were also collected for analysis. Field notes were used as a secondary source of information to provide more in-depth information and support the data obtained from the interviews [43]. All parents were interviewed alone, and interviews were conducted in the Spanish language.

#### 2.2.3. Data Analysis

To analyse the participants’ experience an inductive thematic analysis was performed on the collected data [43,50]. Complete and literal transcriptions were made from each in-depth interview and the researchers’ field notes [43,50]. The thematic analysis approach [43] involved identifying the most descriptive content in order to convert the data into meaningful units and, subsequently, to reduce the data and identify the most common meaningful groups. In this manner, clusters of meaningful units were generated, identifying similar points or content that allowed topics describing the study participants’ experience to emerge [43,50]. This thematic analysis procedure was conducted separately with both the interviews and the field notes. Joint meetings were held to combine the results of the analysis as well as to discuss the data collection and analysis procedures. In the event of differing opinions, theme identification was performed based on a consensus among the research team members. Subsequently, the research team held joint meetings to combine, integrate, and identify final themes.

For the qualitative content statistical analysis, the R Ver. 3.5.1. (R Foundation for Statistical Computing, Institute for Statistics and Mathematics, Welthandelsplatz 1, 1020 Vienna, Austria) was used. The text of the interviews was lemmatized for the analysis. Sentiment analysis was performed using the Bing [54], Afinn and National Research Council Canada (NRC) [55] dictionaries. Likewise, the polarity of the text was analysed using the Bing dictionary, the Semantic Orientation Dictionaries Version 1.11 Spanish [56,57,58] dictionary as amplifiers and decrementators, and those proposed by Vilares et al. [59] as deniers.

Content analysis has been used previously in the health sciences to study medical and patient narratives [60,61]. Four phases were used progressively for the analysis of acceptance-rejection (polarity). First, we created a file with the text of the interviews broken down by phrases for textual analysis. Second, we calculated the polarity using the Bing Sentiment Dictionary [54], the amplifiers and de-amplifiers from SO DictionariesV1.11Spa [56,57,58], and the negators proposed by Vilares et al. [59]. (Table S4). Third, we calculated the scatterplot of the sentences in the text regarding neutrality to identify positive or negative trends. Finally, the evolution of the emotional valence (positive–negative) was presented throughout the interviews. We applied the Fourier transformation to confirm the polarity trend.

### 2.3. Ethical Considerations of the Mixed Methods Intervention

All subjects gave their informed consent regarding inclusion in the study, as well as permission to record their interviews, prior to participating in the quantitative and qualitative studies. The study was conducted in accordance with the WMA Declaration of Helsinki [62], and the protocol was approved by the Clinical Research Ethics Committee at the San Pablo-CEU University.

### 2.4. Integration Procedure (Embedded) for Quantitative and Qualitative Content

In the current study, quantitative and qualitative data were integrated at the method level through embedding one within the other [63], and at the interpretation and reporting level through narrative and joint displays [35,37] (Figure 1). Embedding at the method level occurs in studies with both primary and secondary questions (objectives), when different methods are employed to address each question. In the current study, the implementation of data integration consisted of (a) analysing the primary data set to answer the primary research question (quantitative-intervention), (b) analysing the secondary data set (qualitative), which is embedded within the primary design, and incorporating the secondary results, (c) interpreting how the primary (quantitative) and secondary (qualitative) results answer the quantitative and qualitative questions, and (d) presenting the complete set of findings [35]. Moreover, the narrative approach (contiguous), and joint displays (figures and graphs) were used to interpret and present the findings [35,37]. A contiguous approach to integration involves the presentation of findings within a single report, although the quantitative and qualitative findings are reported in different sections. Integration through joint displays brings the data into a visual medium that enables one to draw new insights beyond what can be gained through the results of the separate quantitative and qualitative methods; for these reasons, we organized the related data into figures, tables, and graphs [37,64].

## 3. Results

The results are reported in the following order: (1) quantitative and intervention results, (2) qualitative results, and (3) mixed method findings (integration) [63].

### 3.1. Quantitative Findings

The sample consisted of 6 participants (5 boys and 1 girl) of the 9 patients selected at the beginning of the study. Three subjects were excluded from the study (2 refused participation, and 1 did not return consent form). The mean age of the children receiving WST intervention was 7.17 years (SD ± 1.60). The mean age of parents was 46.6 years (SD ± 4.5) for fathers and 43 years (SD ± 3.7) for mothers. The levels of ASD severity according to The American Psychiatric Association’s Diagnostic and Statistical Manual of Mental Disorders-5 [65] were 1 (50%), 2 (16.7%), and 3 (33.3%). No session had to be interrupted for safety reasons, and none of the children reported any adverse effects during the sessions.

The PedsQL, PSPCSA, and WOTA 1 results (including descriptive statistics) from the examination before and after the WST intervention are shown in Table 2. Using a Wilcoxon test, the results of physical competence (the subtest of PSPCSA) were found to be significant (*p* = 0.026), and the effect size of the improvement was large (r = 0.64). Two PSPCSA subtests (peer and maternal acceptance) did not indicate significant improvement, but the effect size of values increase was moderate (r > 0.3). Similarly, in 3 of 5 PedsQL scales, the effect size of improvement was large (school functioning, r > 0.5) and moderate (physical and psychosocial health, r > 0.3). Moreover, observed effect size of improvement in aquatic functioning (WOTA 1) was large (r > 0.5).

### 3.2. Qualitative Findings

#### 3.2.1. Results of the Thematic Analysis

We extracted the themes that represented the participants’ experiences through analysing the collected qualitative data, which included interviews and researcher´s notes. Three major themes representing parents’ experiences with WST intervention in children with ASD were extracted from the interviews: (a) the meaning of AT; (b) patterns of behaviour and activities changes, and (c) social communication and social interaction.

(a) The meaning of AT

All participants described AT as a beneficial activity for their children, very much directed towards movement and emphasized the value of it being done together with other children. Parents found it a motivating activity for their children because they love the water and see it as a game. However, they were all confused at the beginning, as they thought it would also include the teaching of swimming.

*“Aquatic therapy as it is a more directed thing because the movement is more… it has a more concrete purpose…”*

(P3)

*“Water is comfortable for him and he likes it…and then because he saw it and enjoyed it as if it was a game (fun).”*

(P4)

*“An activity for children with problems and the ability to work with them in the aquatic environment…many possibilities for many things, mainly teaching of swimming…”*

(P3)

(b) Patterns of behaviour and activities changes

This theme refers to the behavioural changes parents have observed in their children in relation to the AT. All the participants agreed that on the days they go to the pool, the children were more relaxed, happy, serene and calm, showing less insistence on ritualised patterns. Three of the participants described having noticed changes in the routines following AT, decreasing the frustration and being somewhat less inflexible to changes if they occurred out of the pool.

*“Pool day for him is…it changes his face…he is happy…he comes relaxed…very comfortable, with less stereotyped movements.”*

(P1)

None of the participants spontaneously reported changes in hyper/hyporeactivity to sensory input or interests in the sensory aspects of the environment. Four participants indicated that they had no problems with eating and report some improvement in activities in the dressing room, such as their participation in dressing up, although they still needed time and patience. Four participants reported that the teachers from the school have seen improvements in terms of behaviour and the relationship with the other children, observing less disruptive and aggressive behaviour.

*“The teacher has told us that the tantrums and aggressive behaviour have decreased and they last less and less time…”*

(P3)

(c) Social communication and social interaction

This theme describes the skills of the children with ASD on social approach with family and peers, during or after the AT sessions. Three of the participants reported an improvement in parent–child interaction. The remaining participants reported that nothing had changed, and even two of them said that the interaction was still only related to the needs of the children. Similarly, they expressed that the way they relate to their siblings is still to play whatever they want according to their interests, and if this is not the case, they tend to move away, and the deficit in maintaining the play relationship persists. When asked about playing with the peers, all the answers reflected that they usually do not respond when a child proposes to play, or that they only do so if they like the game.

*“In the water he tries to play more with us and tries something more with the other children. He is more attentive and looks for them more often, but it is still only about his specific interest, he still doesn’t approach them if they play something he doesn’t like.”*

(P2)

Regarding the concept of verbal/non-verbal communication, four of the parents stated that they had observed an improvement in non-verbal communication in their children, which allowed them to maintain a certain emotional reciprocity by sharing affects and emotions after the intervention. However, all agreed that with the children in the AT group it is still difficult to develop or maintain the relationship. None of the participants spontaneously reported changes in verbal communication.

*“He looks at us, he looks at us much more when we are in the water with him. He keeps eye contact more often and for a longer time. That calmness helps him to continue connecting with us afterwards, it gives us a certain feeling of connection.”*

(P6)

#### 3.2.2. Results of Emotions and the Acceptance-Rejection (Polarity) Analysis

**Figure 3:**
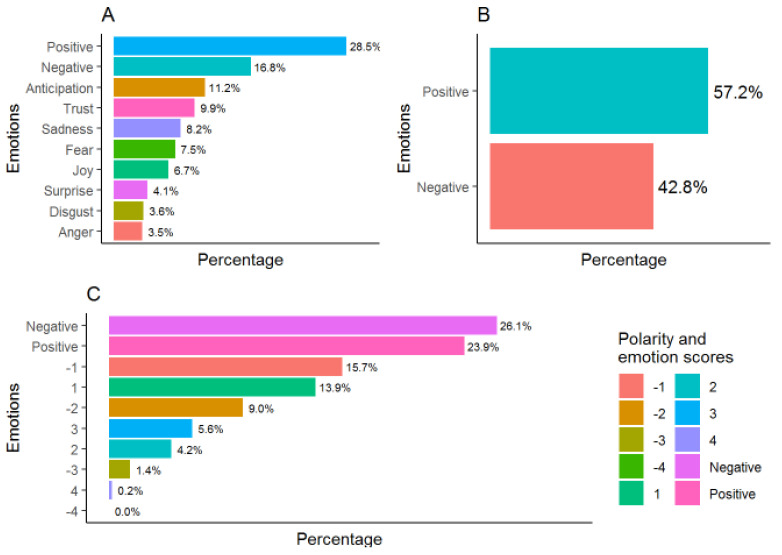
Emotion scores analysed according to National Research Council Canada/NRC (**A**), Bing (**B**), and Afinn (**C**) dictionaries.

The emotions analysis showed a predominance of positive emotions (Figure 3A,B) except in the case of the Afinn dictionary in which negative emotions predominate, especially scores −1 and −2 (Figure 3C). The associated emotions are those of anticipation and trust, followed by the feeling of sadness (Figure 3A).

**Figure 4:**
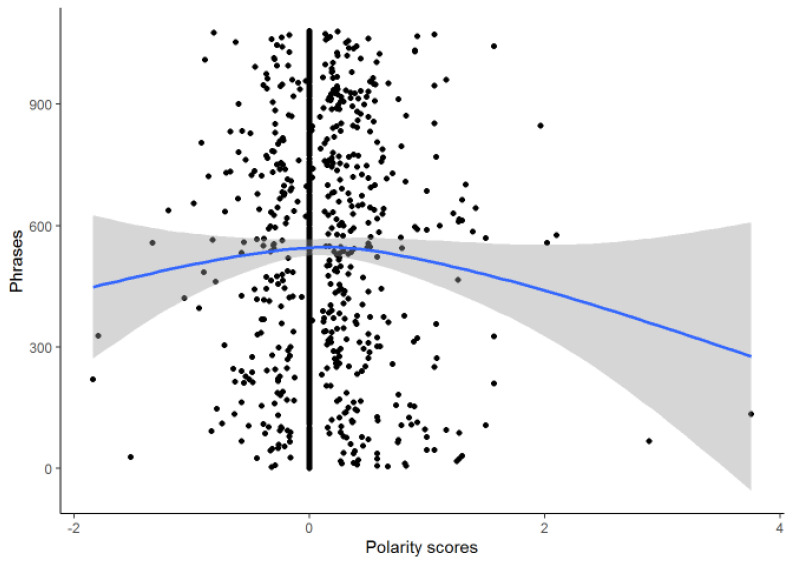
Polarity scores.

The polarity of the interviews was 0.086 ± 0.403 which indicated a discrete trend towards positive emotions due mainly to the presence of extreme positive values (Figure 4).

### 3.3. Mixed Method Findings (Integration)

The results of the integration showed elements of confirmation, expansion, and discordance [37] (Table 3). Our confirmation results demonstrated that the WST intervention including learning strategies led to improvements in physical competence, peer and maternal acceptance, aquatic skills and school functioning. These results were confirmed through the parents’ narratives, which described improvements in childrens’ social interactions and communications post-treatment, at home and at school. Additionally, the content of parents´ interviews reported a predominance of positive emotions and a polarity of acceptance (positive) to the WST intervention.

Our results showed some discordance between quantitative and qualitative results. While the quantitative results presented significant improvement in physical competence, from the parents’ perspective this effect was not relevant. In contrast, parents reported improvements in non-verbal communication, emotional reciprocity, and parent–child interaction.

## 4. Discussion

Our study showed positive results for the social and physical competence, with elements of discordance, expansion, and confirmation between quantitative and qualitative results after the AT intervention. The results provided an expansion of knowledge in the application of the intervention programs for children with ASD and included the parents’ perspective on the intervention. Mixed method design allowed to evaluate the intervention from both clinical perspective and parents’ expectations. Such approach may increase its impact on the functionality and socialisation of the child. Thus far, only few studies have investigated the effect of AT on social interactions and behaviours of children with ASD [24,25], or including learning strategies [19,20,21,22] to achieve certain swimming skills. To our knowledge, no study has described the impact of WST intervention with learning strategies on children with ASD using mixed methods.

Our results revealed statistically significant increase of perceived physical competence and clinically significant improvement in the social acceptance (composed of peer and maternal acceptance). Chu and Pan’s studies showed improved social interactions with peers, siblings, and therapists in all groups during group activity time [24,66]. In both studies, the aquatic interventions were based on the Halliwick concept, resulting in improvements in social interactions and behaviours. Chu and Pan suggested that these improvements may be due to social interaction with peers or siblings, the structure of the aquatic program, and the guidance of the AT instructor [66]. It may also be due to the constant attention they received from the therapist and observations of positive social interactions during the AT sessions [24], which was also noted in our study. In Chu and Pan studies, follow-up monitoring showed that at 10 weeks after the intervention, the positive effect was maintained [24,66]. Vonder Hulls revealed that the most potential benefits of AT included stimulation and vocalisation of language, improved tolerance of physical contact, increased eye contact, and improved self-confidence [67]. These benefits can have holistic effect on relationships with peers and siblings and greater social acceptance, also observed in our study. Chu and Pan suggested that less physical contact with therapists does not necessarily imply a negative outcome. It may improve positive interactions of a child with ASD with the peers [66]. Vonder Hulls et al. presented consistent findings suggesting that a decrease in asking for help from the therapists, by children with ASD, could be treated as a sign that the child is gaining confidence and independence in social interactions, similarly to our results [67].

Learning strategies in our AT program mainly comprised task direction, consecutive steps, prompting, and attentional cues included in the 4 phases of the WST. Among the studies that implemented learning strategies to the aquatic intervention, three used elements of the Halliwick concept (simple progression, water games, and lateral and vertical rotation) [19,20,21] and one [22] used three swimming skills (kick, stroke, and head turns to the side). Rogers’ et al. [22] proposed continuous reinforcement while Yilmaz’s et al. used unique opportunity and social reinforcements, as a feedback during AT. Yilmaz et al. found a decrease in stereotyped movements during the AT session [19,22]. Pan found a decrease in antisocial behaviour and an increase in physical interaction with peers, as well as in social interaction with the peers, the instructor, and another child with autism [24]. In the survey conducted by Volder Hulls et al., more than half of the parents confirmed a significant increase in maintaining eye contact, concentration, and attention in their children [67]. The present study supports such effect of the AT intervention on social acceptance of children with ASD expressed in perceived peer and maternal acceptance.

We observed a clinically significant increase in aquatic functioning after WST intervention, which is in line with authors who showed that children with ASD increased their aquatic skills [19]. Yilmaz et al. revealed improvements in the percentages of errors made and in the total time spent on the achievement of the task. In addition, it was shown that the participants maintained the gains obtained in the first, second, and fourth week after the intervention [19]. Other studies showed an increase in aquatic skills after intervention together with the significant improvement of swimming movements [24,66]. In our study, the AT intervention was based on everyday movements (not swimming) included in aquatic games, and therefore, clinically significant improvement was observed only in aquatic functioning.

The results of this study revealed that quality of life improved in the sub-domains of health aspects and school aspects. Our findings in PedsQL scales showed clinically important improvement in school functioning (large effect size) and in both physical and psychosocial health (moderate effect size). Similarly, Ennis et al. demonstrated that there was an improvement in total scores, social skills, school activities and emotional functioning measured by the PedsQL even with a shorter duration program (10 weeks) than ours [28]. Lack of improvement in social and emotional functioning domains in our study may be related to the appropriateness of the PedsQL for lower functioning children with ASD discussed by Ikeda et al. [68].

Our qualitative results showed a discrete tendency of polarity towards positive emotions with predominance of positive emotions regarding the meaning of AT, patterns of behaviour and activities, and social interactions. Parents’ perception of children’s’ improvements in behavioural changes at the end of the sessions was consistent with previous researches [19,24]. Children were calm and relaxed with a decrease in aggressive and disruptive behaviour. These findings are in accordance with previous authors who found a significant decrease in the antisocial/aggressive and defiant/disruptive behaviours after AT intervention [24]. Yilmaz et al. noted a decrease in stereotyped movements, such as turning, rolling, and delayed echolalia. [19]. Additionally, our results identified improvements in children’s social interactions and communication after treatment, both at home and at school. It should be emphasised that parents highlighted the fun and motivating character of the WST sessions. In all studies [19,24,28], parents noted that their children had fun during therapy in water. None of the studies indicated a negative effect of AT on children with ASD.

Further studies are needed to corroborate the effects of aquatic interventions and to assess the transfer of social interaction outcomes to daily life. In addition, certain requirements must be met during implementation to ensure that these treatments are more effective, also considering the presence and needs of parents during sessions, as well as the social relationships and strategies adopted. These results can inform the development of future AT programs in hospital settings.

The current study has several limitations. The sample size was small, which may demonstrate a lack of statistically significant results, but still provide results with clinical significance. Large and moderate effect sizes obtained in our study for school functioning, physical health, psychosocial health, peer acceptance, physical competence, mental acceptance, and aquatic functioning may be an indicator of future expectations of research results. Nevertheless, future studies should be conducted with more participants. The results of this study should be treated with caution and cannot be generalized to all children with ASD. However, we do not consider this a significant limitation because we used a mixed methods (quantitative and qualitative) approach, with multiple strategies for data collection and analysis, to increase the trustworthiness and credibility of the findings [29,30,35]. Currently, the integration of qualitative and quantitative designs is recommended when evaluating novel interventions for use in health sciences [29,30,35]. Further randomized controlled trials will be required to compare our aquatic protocol with other conventional approaches to learning and to verify our results.

## 5. Conclusions

The WST program, together with specifically designed learning strategies, represents a novel intervention approach that could improve certain social interaction outcomes in children with ASD. However, based on this unique methodology, elements of discordance, extension, and confirmation were identified between the qualitative and quantitative results. It has to be highlighted that mixed method design with embedding quantitative and qualitative data is new for the AT field. Further studies are needed to corroborate the effects of enriched environments intervention combined with conventional therapies for the learning of children with ASD.

The present study has important implications and significance for the development of aquatic programs and interventions based on the use of WST along with learning strategies among children with low-functioning ASD, promoting behavioural changes as the most important outcomes. Our results may help healthcare professionals to better understand children receiving aquatic treatment. The presence of parents during the sessions is an added value for the transfer of children’s learning out of the pool.

## Figures and Tables

**Table 1:** Mixed methods intervention study summary.

Study	Component	Sampling	Participants	Intervention	Data Collection	Analysis
Main study	Water Specific Therapy intervention (non-randomized)	Non-probabilistic sampling of non-consecutive cases	Patients with ASD (diagnostic criteria of DSM-5)	Water Specific Therapy intervention using learning strategies	PSPCSA (perceived competence and social acceptance for young children) WOTA1 (child’s ability to adapt to the aquatic environment and related functional ability), PedsQL (health-related quality of life)	The statistical analysis was performed using the SPSS statistical software system. The Wilcoxon signed-rank test for related samples was used to compare variables. Additionally the effect size was determined by calculating the r
Embedded study	A qualitative case-study	Purposeful sampling and information power criteria	Parents of the participants included in the main study	Non applicable	Semi-structured interviews based on a question guide, and researcher field notes	Thematic inductive analysis was performed. For the qualitative content statistical analysis, the R Ver. 3.5.1. was used. Emotions analysis was performed using the Bing, Afinn, and NRC dictionaries. Likewise, the polarity of the text was analyzed using the Bing dictionary, the SO Dictionaries V1.11 Spa dictionary

PedsQL—The Pediatric Quality of Life Inventory, PSPCSA—the Pictorial Scale of Perceived Competence and Social Acceptance for Young Children, WOTA 1—the Water Orientation Test Alyn version 1, DSM-5—The American Psychiatric Association’s Diagnostic and Statistical Manual of Mental Disorders-5, NRC—National Research Council Canada. SO Dictionaries V1.11 Spa—Semantic Orientation Dictionaries Version 1.11 Spanish.

**Table 2:** Results of quantitative evaluation of the WST intervention for children with Autism Spectrum Disorder (*n* = 6).

Variable			Mean (SD)	Min	Max	*p*-Value ^1^	Effect Size r
PedsQL	Total score	Pre	64.11 (23.79)	33.91	88.44	0.249	0.333
		Post	69.77 (18.90)	40.00	90.47		
	Physical Health	Pre	69.79 (32.46)	18.75	96.88	0.206	0.365
		Post	76.56 (26.86)	25.00	96.88		
	Psychosocial Health	Pre	62.22 (22.20)	31.67	86.67	0.207	0.364
		Post	67.50 (17.22)	45.00	88.33		
	Emotional Functioning	Pre	63.33 (18.07)	35.00	90.00	0.357	0.266
		Post	66.67 (15.38)	45.00	90.00		
	Social Functioning	Pre	70 (23.24)	40.00	100.00	0.343	0.274
		Post	74.17 (20.60)	50.00	100.00		
	School Functioning	Pre	53.33 (31.41)	15.00	95.00	0.066	0.531
		Post	61.67 (24.22)	30.00	95.00		
PSPCSA	Cognitive competence	Pre	15.33 (7.89)	6.00	24.00	1.00	-
		Post	15.33 (7.89)	6.00	24.00		
	Peer acceptance	Pre	14.83 (4.62)	10.00	23.00	0.257	0.372
		Post	15.33 (4.18)	12.00	23.00		
	Physical competence	Pre	13.00 (4.34)	7.00	18.00	0.026 *	0.644
		Post	14.83 (4.17)	9.00	20.00		
	Maternal acceptance	Pre	17.83 (1.17)	17.00	20.00	0.157	0.408
		Post	18.17 (1.47)	17.00	21.00		
WOTA 1		Pre	46.50 (7.29)	33.00	52.00	0.066	0.531
		Post	48.50 (6.25)	36.00	52.00		

* *p* ≤ 0.05; ^1^ significance of absolute value of T statistic (Wilcoxon test); PedsQL—The Pediatric Quality of Life Inventory, PSPCSA—the Pictorial Scale of Perceived Competence and Social Acceptance for Young Children, WOTA 1—the Water Orientation Test Alyn version 1, WST—Water Specific Therapy.

**Table 3:** Combined display of the quantitative and qualitative findings.

Outcomes	Quantitative Findings	Qualitative Findings	Observations
PSPCSA: measures the perceived competence and social acceptance	Statistically significant improvement in physical competence with large effect size Clinically significant improvement in peer and maternal acceptance with moderate effect size	There was no narrative regarding movement improvement in daily-life activities Parents observed an improvement in non-verbal communication in their children, which allowed them to maintain a certain emotional reciprocity by sharing affects and emotions after the intervention Participants reported improvement in parent–child interaction	Parents believed the WST intervention was aimed at learning swimming, although later parents reported improvements in the aquatic skills related to daily life. Parents reported their experiences with the WST, describing it as a motivating intervention for their children, which made them feel comfortable as well. Through the sensory adaptation and structured play strategies, they saw the children enjoying themselves. This process resulted in satisfaction with the perceived changes. Polarity results showed a positive emotions with respect to the application of WST, although some parents pointed out the necessity of more clear explanation the non-swimming goals.
WOTA1: measures the child’s ability to adapt to the aquatic environment and related functional ability	Clinically significant improvement in aquatic functioning with large effect size	Parents reported improvement in aquatic movement games developed in groups	
PedsQL: measures health-related quality of life	Clinically significant improvement in school functioning (large effect size), and both physical and psychosocial health (moderate effect size)	Participants reported that the teachers from the school have seen improvements, in terms of behaviour and the relationship with other children, observing less disruptive and aggressive behaviour	

PedsQL—The Pediatric Quality of Life Inventory, PSPCSA—the Pictorial Scale of Perceived Competence and Social Acceptance for Young Children, WOTA 1—the Water Orientation Test Alyn version 1; WST—Water Specific Therapy.

## Data Availability

Not applicable.

## Supplementary Materials

The following are available online at https://www.mdpi.com/1660-4601/18/6/3126/s1, Table S1: Template for intervention description and replication (TIDieR) checklist. Table S2: The applied trustworthiness qualitative design criteria. Table S3: Semi-structured question guide. Table S4: Polarity analysis.
