# Supplementary material for: Effects of Aquatic Therapy for Children with Autism Spectrum Disorder on Social Competence and Quality of Life: A Mixed Methods Study
Source: Int J Environ Res Public Health. 2021 Mar 18;18(6):3126. doi: 10.3390/ijerph18063126 (PMC8002945; doi:10.3390/ijerph18063126)
Supplement: Supplementary file 1 [file ijerph-18-03126-s001.pdf]

## Supplementary materials.

**Table S1.** The Template for Intervention Description and Replication (TIDieR) Checklist [40].

| | |
|---|---|
| **1. Brief name** | Water Specific Therapy-Halliwick (WST) with learning strategies in children with Autism Spectrum Disorder (ASD) for improving social competence and quality of life: A mixed method intervention study. |
| **2. Why** | ASD are multifactorial disorders of neurodevelopment that present significant impairment in social communication and repetitive sensory and motor behaviours. The aim of this study was to implement an aquatic therapy (AT) program WST-Halliwick, using learning strategies specifically designed by the research team for children with ASD. Furthermore, we sought to analyse its effects on perceived competence and social acceptance, aquatic skills and quality of life. |
| **3. What material** | A WST session consists of four different phases:<br>1. In the first phase (entry ritual) the approach to the new environment (as social engagement) has to be stimulated. It is necessary to be very methodical and to follow clear routines for anticipation. At home, the aquatic therapy session can be introduced by means of a communication board using pictograms or real images of the pool, or by carrying out a programme to make a backpack. A group gathering can also be organized at the entrance. Once in the pool, entry into the water can be announced by pictures or pictograms, stories, song association or group games.<br>2. In the second phase (mental adjustment) the water begins to be experienced as a sensorimotor environment, as a way for the child to enter it through play. This is the phase of the first aquatic perceptions, and it is the child who decides what to explore and how. This is why it is a phase of a certain initial independence (disengagement from the therapist and the task) after having achieved their engagement to the aquatic environment in the first phase. Therapists must follow the child (joint attention, requests, imitations), to allow a close relationship between the two (again, engagement) and motivate the child to continue discovering, but never force him/her; go at the pace set by the child, to avoid rejection, lack of control or dysregulation. Gradually make this process more structured. It is a perceptual stage in which, without giving a clear guideline at the beginning and leaving the control of the session to the child at first, the child explores through activities such as splashing, sinking and pouring, filling and emptying, or moving away and approaching. The mission is to wait, to observe, to ask for permission, to interact with the child in a gradual way. The aim for the child is to connect with the environment and with the therapist (social engagement).<br>3. The main phase (learning) builds on the previous phases by creating rules and norms based on what the child has previously decided to explore. Here the WST offers a multitude of opportunities for interaction, from the 10 points, through play options that are added, as well as all the pedagogical and learning tools that are extremely versatile in this aquatic dynamic. In this phase, in its social aspect, we can again vary between dependence and independence with respect to the therapist, the task, the environment and the family (engagement or disengagement). This is the phase in which we seek, through motor and mental work, to change the behaviour of the children with ASD, as well as to try to consider the transfer of all that has been learnt to their daily life. In order to do this, it is important to design specific tasks for each child, which present a clear and adjusted goal. During the execution of each task we should use strategies that |

| | |
|---|---|
| | support learning, such as visual aids, positive reinforcement, trial without error, active work and repetition. Apart from using these strategies, during the session we must control and dose what kind of sensory inputs we give to the child to enable an optimal state of alertness. It makes normal development possible during the session, and in turn facilitates the acquisition of new social skills and behavioural change.<br>4. In the last phase (exit ritual) our aim is that the child calms down and connect with his/her transfer out of the water. Creating a routine that informs them of the time of the exit and the activities that will take place afterwards contributes to a better performance of the activity we are asking them to do (saying goodbye to the water). We can do this through songs or imitation games, or simply by putting the toys away, accompanied by the use of pictures which is often effective in reducing anger and tantrums at the end of the session. We never deny the emotional states of the child (any emotion is welcomed and it is important to name it and collaborate to ensure that the child acquires skills for self-regulation). |
| **4. What procedures** | 1. Ritual of entry. Routine phase. Structure the entry:<br>• Songs, stories, tales, rules, visual agenda.<br>2. Mental adjustment + control of WST-Halliwick rotations. Perceptual phase. Explore water by themselves. Link with therapist who respects and follows initiative:<br>• 1-1/group Therapeutic exercises/games. Mental-motor independence: Encourage regulation at all times. Exaggerate emotions and gestures to maintain interaction. Little by little give structure (rhythm).<br>• Safety exercises: Balance, falls, turns, etc.<br>• Presymbolic play: Splashing, Cause-effect, Go-come, Throwing and picking up games, Space permanence, Appearance-disappearance, Object permanence, Sinking-out, hiding and finding.<br>• Functional/symbolic play: "As if" with real objects, Threading more actions into the "as if" play and suspending the real use of objects, Using invisible objects and characters.<br>3. Learning + WST control rotations. Conceptual phase. Goal setting:<br>• Begin to change behaviour and transfer.<br>• 1-1/group play.<br>• Select a Halliwick point to do the rehearsal-learning. Use necessary supports, modelling, chaining. Structure sensory inputs and sequenced tasks. Add learning aids (reinforcers, visual aids, etc.).<br>4. Exit ritual. Routine phase.<br>• Calming down. Structure the exit with activities that organise or regulate.<br>• Songs, picking up toys or elements. Use goodbye songs, songs to say goodbye. Use farewell songs, pictograms diary, songs, collecting toys or material. |
| **5. Who provided** | In water, ideation (mental desire) and motor control go together. Play is the perfect opportunity to link the two within the aquatic intervention process, as a source of fun, motivation and learning. A great advantage for the quality of the intervention is that the family can be included in the session. It can result repetition and generalisation of the games and therapeutic goals in their natural environments, even improving the self-concept and sense of security of the parents.<br>The WST games were developed by 2 physical therapists with more than 10 years of expertise. Two evaluators (physical therapists) with more than 10 years of expertise performed the initial and post-treatment assessments. AT Sessions were |

| | |
|---|---|
| | carried out by one physical therapist who were with the patients throughout all the rehabilitation process. |
| **6. How** | Group sessions. Attended by the 6 children, the physical therapist and the childrens´ father/mother who participated in WST activities. |
| **7. Where** | The intervention took place in a therapeutic pool:<br><br>- 1.10 m depth<br><br>- Water temperature 31.5 ºC<br><br>- Access to therapeutic area via a ramp or staircase<br><br>- Water quality: pH and chlorine measurements were performed twice a day. The rest of the parameters were checked daily by the maintenance personnel. The pool water was constantly filtered (overflow) and all the water was renewed daily. |
| **8. When and how much** | Patients attended AT twice a week over seven months. Sessions lasted 60 minutes, including a change of clothes, physiotherapy session and free time with parents. The day of therapy and the schedule was agreed on with the parents depending on the best availability to attend other therapies, medical consultations and adapted to school hours. Moreover, we tried to schedule therapy in time slots when the pool occupancy is less than 25% to avoid noise and crowds. |
| **9. Tailoring** | During the sessions different play strategies based on circular play models were used, in which the child is helped to move towards higher levels of complexity with strategies to facilitate social interaction. The child's interests are respected and noted, as internal motivation helps to improve the quantity and quality of joint attention. This facilitates the establishment of WST games in which the child has to overcome challenges (making requests, taking turns or playing in a team).<br><br>One recommendation when starting a play stimulation program in the aquatic environment is to let the child lead at first, with us intervening in a gradual and orderly manner. The aim is to connect their actions within a pedagogical approach that will help us to attract their attention, to get them to accept us within their space and to engage them emotionally. A good tip at the beginning is the use of counter-mimicry, observing the child's intentional actions. Other strategies include setting some kind of problem to solve, hiding a toy, making a funny mistake or to using elements in unconventional ways. |
| **10. Modifications** | There were no modifications of the initial protocol. All patients were able to perform all the proposed activities. |
| **11. How well planned** | Intervention was delivered as planned and no adverse side-effects were observed. Physical therapist registered children attendance to sessions through a name check sheet. |
| **12. How well actual** | Intervention was delivered as planned and no adverse side-effects were observed. |

**Table 2.** Trustworthiness qualitative design criteria applied.

| Criteria | Techniques performed and application procedures |
|---|---|
| Credibility | Investigator triangulation: each interview was analyzed by two researchers. Thereafter, team meetings were performed in which the analyses were compared and themes were identified.<br>Triangulation of data collection methods: semi-structured interviews were conducted and researcher field notes were kept. |
| | Participant validation: this consisted of asking the participants to confirm the data obtained during the data collection and analysis stages. |
| Transferability | In-depth descriptions of the study were performed, providing details of the characteristics of researchers, participants, contexts, sampling strategies, and the data collection and analysis procedures. |
| Dependability | Audit by an external researcher: an external researcher assessed the study research protocol, focusing on aspects concerning the methods applied and study design. |
| Confirmability | Investigator triangulation, data collection triangulation.<br>Researcher reflexivity was encouraged via the completion of reflexive reports and by describing the rationale for the study. |

**Table 3.** Semi-structured question guide.

| Research topics | Questions asked |
|---|---|
| Opening questions | What does aquatic therapy mean to you? What is your treatment expectation?<br>What is your experience with the aquatic therapy received by your child? |
| Adaptation to the new aquatic environment for WST treatment | How was your child's adjustment to the new treatment environment? Were there any activities that were very difficult to perform? |
| Effects of WST treatment | How do you think the aquatic therapy has influenced your child? What do you think has been the benefit of participating in these sessions?<br>Have the recommendations provided by the aquatic therapists been useful for the management of your child outside the pool? |
| Influence of aquatic therapy on the daily care and management of the child | In what aspects of daily care and management of the child have you noticed changes? If these were negative, what were they? |
| Factors of the disorder for which aquatic therapy is most useful | How do you feel aquatic therapy can affect your child in the long term? |

WST: water specific therapy

**Table S4**. Polarity analysis

| Formula and dictionaries used to calculate polarity |
|---|

- The analysis was carried out using the Bing dictionary [54]. The Bing dictionary determines the positivity (acceptance) or negativity (rejection) of each word used. Also, the amplifiers and de-amplifiers of Semantic Orientation Dictionaries Version 1.11 Spanish dictionary [56-58] were used, and the negators proposed by Vilares et al. [59].
- To calculate the polarity (δ), a contex cluster of words ($x^{T}_{i}$) was formed around each polarized word using the Bing dictionary [54], taking by default 4 words before and two words after it (if there is any comma in the cluster, it will only include the words that are after the comma) and that will be treated as valence shifters.
- The words in this cluster are labeled as neutral ($x^{0}_{i}$), negators ($x^{N}_{i}$), amplifiers ($x^{a}_{i}$) or de-amplifiers ($x^{d}_{i}$) using the dictionary SODictionariesV1.11Spa2 [56-58] and the negators proposed by Vilares et al. [59]. Neutral words do not add to the equation but affect the word count (n).
- Each polarized word (negative or positive) is weighted (w) based on the context cluster weights ($x^{T}_{i}$) and further weighted by the number and position of the valence shifters directly surrounding it. A weight (c) can be added and applied to both amplifiers and de-amplifiers (with a default value of 0.8 and a lower limit for the de-amplifiers of -1).
- Finally, the context cluster ($x^{T}_{i}$) is added and divided by the square root of the number of words (√n) to generate a polarity score (δ) that by default is not limited in value.

---

The end result is the formula:

$$\delta = x^{T}_{i} / \sqrt{n}$$

---

Where:

$$x^{T}_{i} = \sum ((1 + c(x^{A}_{i} - x^{A}_{i})) \cdot w(-1)^{\sum xNi})$$
$$x^{A}_{i} = \sum (w_{neg} \cdot x^{a}_{i})$$
$$x^{D}_{i} = \max (x^{D'}_{i} - 1)$$
$$x^{D'}_{i} = \sum (-w_{neg} \cdot x^{a}_{i} + x^{d}_{i})$$
$$w_{neg} = (\sum x^{N}_{i})$$

---
